# Factors associated with loss to follow-up among people with tuberculosis in the country of Georgia: a cohort study

**DOI:** 10.4081/monaldi.2021.1705

**Published:** 2021-01-14

**Authors:** Natalia Adamashvili, Kristina Akopyan, Nestani Tukvadze, Kostyantyn Dumchev, Yuliia Sereda, Irma Khonelidze, Giorgi Kuchukhidze

**Affiliations:** 1 National Center for Disease Control and Public Health, Tbilisi, Georgia; 2 Tuberculosis Research and Prevention Center NGO, Yerevan, Armenia; 3 National Center for Infectious Diseases, Yerevan, Armenia; 4 National Center for Tuberculosis and Lung Diseases, Tbilisi, Georgia; 5 Ukrainian Institute of Public Health Policy, Kyiv, Ukraine; 6 World Health Organization, Regional Office for Europe, Copenhagen, Denmark

**Keywords:** Tuberculosis, pulmonary tuberculosis, loss to follow-up

## Abstract

Despite having universal access to tuberculosis (TB) treatment, loss to follow-up (LFU) rates remain high in Georgia, 6% among drug-susceptible TB (DS-TB) patients (2017 cohort) and 19% among drug-resistant TB (DR-TB) patients diagnosed in 2016. A cohort study was conducted to analyze secondary data from the Georgian National Tuberculosis Surveillance Database. Study population included adult (≥18 years) patients with bacteriologically confirmed pulmonary TB who were enrolled in Georgian National TB program during 2015–2017. The outcome of interest was loss to follow-up, defined as treatment interruption for more than 2 consecutive months. Patients were stratified by treatment profile (first-line drugs or second-line drugs) and survival analysis was performed within the stratified groups. A total of 7860 treatment episodes were identified during 2015–2017 which corresponded to 6696 bacteriologically confirmed pulmonary TB treatment episodes of whom 795 (12%) were LFU. After adjustment, final multivariate analysis showed that male sex (aHR 1.5, 95% CI 1.2–2.0), being diagnosed in Tbilisi (aHR 1.3, 95% CI 1.1–1.6), unemployment at the time of diagnosis (aHR 1.7, 95%CI 1.2–2.3) and previous history of TB treatment were independent risk factors for LFU (aHR 2.3, 95% CI 1.9–2.8) among patients on first-line drugs. Among patients on second-line drugs being male (aHR 2.0, 95% CI 1.2–3.2), past TB treatment with second-line drugs (aHR 2.2, 95% CI 1.5–3.2) were significantly associated with LFU. LFU rate was high among patients on first-line drugs and second line drugs (10% and 22% respectively). Patients with past TB treatment history should further research to identify factors that lead to treatment interruption in this group. Other factors associated with LFU (being internally displaced person (IDP), being unemployed, and having imprisonment history) were in some level indication of a poor social-economic status and strengthening approaches for TB care based on patients’ need could be considered in light of this finding.

## Introduction

Tuberculosis (TB) remains a major public health problem worldwide. In 2019, World Health Organization (WHO) reported an estimated 10.0 million incident TB cases worldwide. About half a million new cases of TB were rifampicin-resistant or multi-drug resistant (RR/MDR-TB). There were an estimated 1.2 million TB deaths among HIV-negative people and 251,000 deaths among people living with HIV (PLHIV) [1,2].

Loss to follow-up (LFU) remains a barrier for TB control and contributes to low treatment success rate globally. In 2020 in WHO European region LFU rate was 4.6% for new and relapse TB patients on first line drugs (FLD), with the highest rates reported in Armenia (13%), Czech Republic (11.7%), Belgium (10.9%), Kyrgyzstan (10.3) and Hungary (8.8) [3].

LFU is a barrier for TB control especially among patients on second line drugs (SLD) [1]. TB treatment with second line drugs requires longer duration and is often accompanied with side effects. Because of these factors, patients often interrupt treatment [4]. Globally, percentage of patients with drug-resistant TB (DR-TB) who started treatment in 2016 and who were successfully treated (cured or completed treatment outcomes) was 56% and the rate of LFU was about 15% (with highest rate reported in the WHO Region of the Americas, 25%).^1^ Several studies have described factors associated with LFU, including being male, having side effects, being previously treated for TB, drug abuse and having HIV infection [5–8]. These studies mostly focus either on patients with first line drugs or second line drugs or did not have treatment regimen included as a factor being associated with LFU.

Despite having universal access to both drug-susceptible and drug-resistant TB treatment, LFU rates remain high in Georgia, especially among patients with DR-TB. A study conducted among DR-TB patients who received treatment from 2009 to 2011 showed that rate of LFU was as high as 29% [8]. Last few years show a declining trend of LFU rates in Georgia; however, the rate still remains high, reaching 6% among drug susceptible TB patients on first line drugs (2017 cohort) and 19% among TB patients on second-line drugs (2016 cohort) (*unpublished data*, from Georgian National Tuberculosis Program Surveillance Database). LFU also contributes to high TB mortality in the country. A research carried out by the National Center for Disease Control and Public Health (NCDC) Tbilisi, Georgia and the National Center for Tuberculosis and Lung Diseases (NCTLD) showed high proportions of death (23%) in patients who started second-line treatment and were LFU during 2011–2014 [9]. To our knowledge, factors associated with LFU have not been assessed to recent TB cohorts in terms of treatment profile. In addition, now that new drugs were introduced, studying recent TB cohorts could determine whether there is difference before and after new drugs. Our study aimed to evaluate the factors associated with LFU among adult patients with bacteriologically confirmed pulmonary TB who were enrolled in National Tuberculosis Program during 2015–2017 in the country of Georgia. The study focused on socio-demographic, disease- and TB treatment-related factors that could be associated with LFU.

## Materials and Methods

### Study design

We conducted a cohort study analysing secondary data from the Georgian National Tuberculosis Surveillance Database.

### Study setting

#### General:

Georgia is an upper middle-income country located in eastern Europe with estimated 3.7 million population [10].

#### TB Program:

Georgia has universal coverage for TB care since 2009. National Tuberculosis Program (NTP) is implemented and administered by head facility for TB control - National Center for Tuberculosis and Lung Diseases (NCTLD) [11]. Georgia has national laboratory network countrywide using molecular diagnostics since 2017. Before rapid tests were introduced, DR-TB diagnosis was based on drug susceptibility testing (DST). All patients receive directly observed treatment (DOT) in TB facilities throughout the country in line with WHO treatment guidelines. In July 2015, the country started using new TB drugs (i.e., bedaquiline and delamanid) for DR-TB within the NTP [12].

### Study population / participants

Study population included adult (≥18) patients with bacteriologically confirmed pulmonary TB who were enrolled in Georgian National TB program during 2015–2017. Bacteriological confirmation included TB diagnosed either one of the following tests: sputum microscopy, GeneXpert or culture. If at least one of the tests was positive, treatment episode was considered to be bacteriologically confirmed.

### Data variables

The outcome of interest was loss to follow-up (LFU), defined as treatment interruption for more than two consecutive months [2]. Data was extracted from the Georgian National TB Surveillance Database. Variables included: i) Demographic information: age, sex, region, marital status, employment status, being an internally displaced person (IDP), body mass index (BMI), tobacco use, alcohol intake, lifetime history of intravenous drug use and imprisonment history; ii) Clinical characteristics: past TB history, type of TB treatment (first line drugs, second line drugs), initial sputum smear result and culture result, drug resistance type (RR/MDR, XDR), HIV and HCV test results, diabetes. Treatment duration was calculated from date of the treatment initiation to date of the treatment outcome.

Some of the variables were available only for patients on second line drugs, as for patients treated with second-line drugs more information is recorded. These variables included: marital status, BMI, tobacco use, alcohol intake, intravenous drug use and diabetes. All other variables were available for all patients.

### Statistical analysis

Data was de-identified and analysed using IBM SPSS Statistics v.23 and R software v. 4.0.2. Before the analysis, data was cleaned and records that did not meet inclusion criteria were removed. We summarized sociodemographic and clinical characteristics of the study participants with frequencies and proportions (for categorical variables) and mean (and standard deviation) or median (and interquartile range) for continuous variables, as appropriate.

Patients were stratified by treatment profile (first line drugs or second line drugs) and survival analysis was performed within the stratified groups. Person-time was calculated from date of the treatment initiation to date of the treatment outcome. We used Cox proportional hazards models to calculate crude and adjusted hazard ratios (cHR and aHR) and 95% confidence intervals (CI) for factors associated with LFU. LFU was compared to favourable outcome (cured or completed) whereas death and treatment failure were treated as competing risks. In the multivariable models we included variables that showed significant result in crude analysis at p<0.1. Proportional hazard assumption was verified for each variable in the final model.

## Results

### Study population

**Figure 1.**
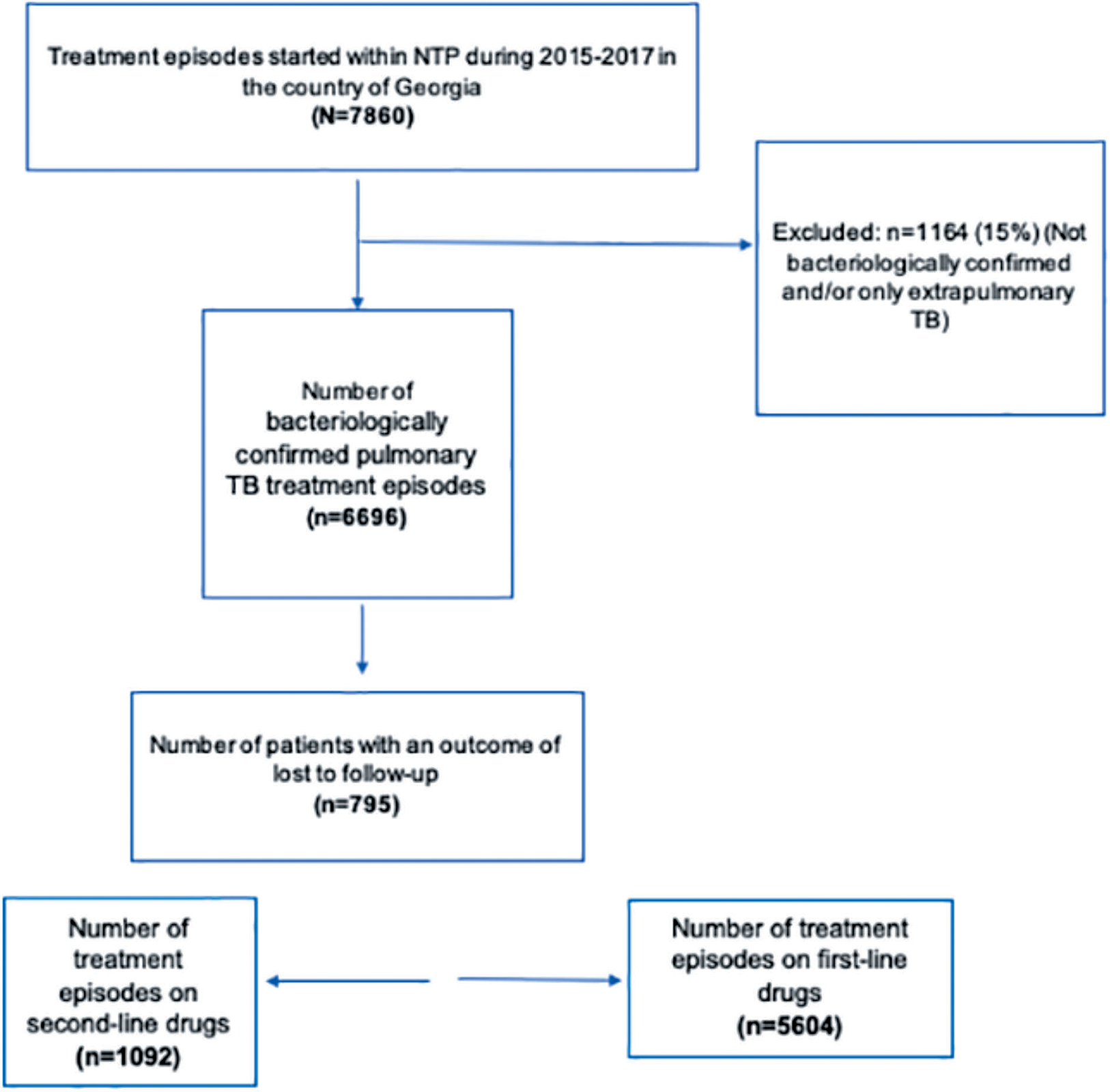
Flowchart of bacteriologically confirmed pulmonary TB treatment episodes from Georgia who were enrolled in National TB Program during 2015–2017.

A total of 7860 treatment episodes were registered at the NTP database in the period of 2015–2017, which corresponded to 6696 bacteriologically confirmed pulmonary TB treatment episodes, of whom 795 (12%) were LFU (Figure 1).

Majority of study population received first line drugs (5604/6696, 84%), remaining 16% (1092/6696) received second line drugs. Median age was 43 years among patients on FLD and 39 among patients on SLD, 76% (5072/6696) of patients were male. With regard to TB treatment history, almost one-third of patients in the total sample (2033/6696, 30%) had been treated for TB in the past. Drug resistance profile in the total sample was the following: 15% (984/6696) of the patients had RR/MDR-TB out of whom 39% (380/984) were pre-XDR or XDR. In the total sample, more than a third of the patients were from the capital city Tbilisi (2407/6696, 36%) and 3% (231/6696) of patients were imprisoned at the time of the diagnosis, majority of the patients were unemployed (5670/6696, 85%). In the total sample about 9% had history of imprisonment out of those for whom the data were available (n=586/6319). Among patients whose HIV status was known, 3% (n=170/5696) were HIV-positive at the time of diagnosis, HCV test result was available for 677 SLD treatment episodes, out of which 202 (30%) were positive. Among patients on SLDs 10% (107/1092) had diabetes, 57% were smokers (617/1092), 42% had moderate or excessive alcohol intake (462/1092) and 3% had history of intravenous drug use (38/1092) (Table 1).

### Factors associated with LFU among patients on first line drugs

In crude analysis, being male (cHR 1.6, 95% CI 1.3–2.0), having history of imprisonment (cHR 1.8, 95% CI 1.3–2.3), being internally displaced person (IDP) (cHR 1.7, 95% CI 1.3–2.3), being in prison at the time of diagnosis (cHR 0.4, 95%CI 0.2–0.9), being unemployed (cHR 1.8, 95%CI 1.3–2.5) and having past TB treatment history (cHR 2.6, 95% CI 2.2–3.1) were associated with LFU (Table 2).

The multivariable analysis showed that male sex (aHR 1.5, 95% CI 1.2–2.0), being diagnosed in Tbilisi (aHR 1.3, 95%CI 1.1–1.6), being unemployed at the time of diagnosis (aHR 1.7, 95% CI 1.2–2.3), being IDP (aHR 1.6, 95% CI 1.2–2.2), imprisonment history (aHR 1.4, 95% CI 1.0–1.8) and previous history of TB treatment (aHR 2.3, 95% CI 1.9–2.8) were independent risk factors for LFU (Table 2).

### Factors associated with LFU among patients on second line drugs

Crude analysis among patients on SLDs showed that male sex (cHR 2.3, 95% CI 1.5–3.6), imprisonment history (cHR 1.6, 95% CI 1.2–2.2), being HIV positive (cHR 2.0, 95% CI 1.2–3.3), moderate alcohol intake (cHR 1.4, 95% CI 1.0–1.8), having diabetes (cHR 0.5, 95% CI 0.3–0.9), being HCV positive (cHR 2.0, 95% CI 1.4–2.7), previous treatment history with first line drugs (cHR 1.5, 95% CI 1.1–2.1) and second line drugs (cHR 2.0, 95% CI 1.5–2.8) were associated with LFU (Table 3).

In multivariable models, being male (aHR 2.0, 95% CI 1.2–3.2) and having past TB treatment history with second line drugs (aHR 2.2, 95% CI 1.5–3.2) were independent risk factors for LFU (Table 3).

## Discussion

Our study found that 12% of patients with bacteriologically confirmed pulmonary TB who were enrolled in NTP during 2015–2017 were lost to follow-up. The proportion is higher compared to the average 5% LFU rate in WHO European Region [3]. Additionally, LFU rate was different between patients on first line drugs and second line drugs (10% and 22% respectively), this difference is in line with previous publications. A study conducted in Haiti showed that 9% of drug-susceptible treatment episodes were LFU during treatment [7]. Study conducted in Pakistan among patients with MDR-TB reported LFU rate 18.3% [13], and another study in South Africa among patients with RR/MDR-TB reported 16% LFU rate at the 12 months of treatment [14].

In our study, one of the factors being an independent risk factor for LFU was male sex both for patients receiving first line drugs (1.5 times greater risk) and second line drugs (1.74 times greater risk). Male sex being a risk factor have been reported in previous studies. A study conducted in Cape Town also reported being male as a risk factor for LFU (aOR 1.3) [15]. Study participants included only adolescents and young adults (10–24 years old) with DS-TB who were on first line drugs and did not include patients on second line drugs. Another study conducted in Georgia evaluating patients with MDR-TB also reported an association between being male and LFU (aHR 1.4) [8]. Another study conducted in Tajikistan, that evaluated LFU risk factors among patients on first-line drugs also found male sex being associated with LFU (cOR 1.78, 95% CI 1.33–2.40) [6]. Study conducted in Nairobi, Kenya reported male sex being a risk factor for LFU and suggested that possible explanation could be that males in area of Nairobi are daily workers and have to choose between working or going to clinic to take medications.

Another factor common for both groups that was associated with LFU was past TB history. This result is consistent with previous publications. A study conducted in Tajikistan revealed that those who had history of previous TB treatment have about twice higher odds of LFU (aOR 2.03, 95%CI 1.05–3.93) [6]. In a study conducted in Cape Town adjusted odds ratio for the same factor was 3.2 [15] and in Georgia 1.35 [8]. This could be an indication that among patients who often interrupt treatment, being less adherent could be caused by other factors that may be common to these patients.

This study also identified factors specifically associated with treatment regimen. Being diagnosed in Tbilisi was another risk factor among patients on first-line drugs. These may be due to the reason that patients living in regions often come to capital city for diagnosis and then continue treatment in regional TB facilities. Thus, this characteristic does not necessarily indicate that people who live in Tbilisi are at higher risk of being LFU.

Other factors associated with LFU among patients on FLDs were having been an internally displaced person (IDP), unemployment and imprisonment history. In Georgia, a person is defined as internally displaced if they had to move from their permanent living place due to war. These people mostly come from Abkhazia, Georgian region that they had to leave during a war in early 90s. Information about being IDP is recorded during the TB diagnosis and is self-reported. Patients who are IDP may have lower income and poor social-economic status that may lead to treatment interruption. The same could be applied to patients who were unemployed and had imprisonment history, as mostly these patients have low or no income and poor socio-economic status. Other studies also report poor socio-economic status as a factor associated with treatment interruption. Study in Estonia among culture-confirmed pulmonary TB patients found unemployment and imprisonment history to be a risk factor of LFU [16]. Another study in South Africa found that steady employment was associated with decreased risk of LFU [17].

Our study has several limitations. First, we used retrospective secondary data from the database that may have errors during data entry process. Second, some of the variables recorded into the database are self-reported and may have been underreported. Third, information on treatment categories (i.e., first line and second line drugs) were based on DST profile and/or information on treatment regimens. In the database, DST results and treatment regimens are recorded at the beginning of the treatment but may change during the treatment episode which is not being captured in the database.

Our study has following strengths: The National TB Program covers the entire country including all TB patients who were enrolled in TB program during the study period. Thus, study results are representative and generalizable to entire Georgia. Another strength is a large sample size that allows to conduct adjusted analysis, increasing the estimates’ precision.

Our study found that rates of LFU remain extremely high among patients on SLD (22%). Even though LFU rates among patients on FLDs are lower compared to patients on SLDs, it is still high (10%) compared to WHO European region average [3]. Further qualitative research could identify reasons for treatment adherence and interruptions, which could guide targeted interventions by the NTP. In addition, further research is needed among patients with past TB history to determine and evaluate factors that lead to LFU in this group. Other factors that were associated with LFU were all in some level indication of a poor social-economic status (being internally displaced person (IDP), were being unemployed and having imprisonment history). Thus, strengthening approaches for provision of care based on patients’ needs could be considered in light of this finding.

## Figures and Tables

**Table 1.** Socio-demographic and clinical characteristics of bacteriologically confirmed pulmonary treatment episodes enrolled in NTP during 2015–2017 in the country of Georgia.

Characteristics	Total N=6696(%)	Patients on FLDs n=5604 (%)	Patients on SLDs n=1092 (%)
Gender			
Male	5072 (76)	4182 (75)	890 (81.5)
Female	1624 (24)	1422 (25)	202 (18.5)
Age			
18–30	1516 (22.6)	1223 (22)	293 (27)
31–45	2189 (32.7)	1782 (32)	407 (37)
46–65	2326 (34.7)	1976 (35)	350 (32)
>65	665 (9.9)	623 (11)	42 (4)

Region			
Tbilisi	2407 (36)	1656 (30)	751 (69)
Other	4289 (64)	3948 (70)	341 (31)
Employment			
Employed	783 (12)	647 (12)	136 (13)
Unemployed	5670 (88)	4764 (88)	906 (87)

IDP history			
Yes	430 (6)	367 (6)	63 (6)
No	5753 (86)	4922 (88)	831 (76)
Unknown	513 (8)	315 (6)	198 (18)
Imprisonment history			
Yes	586 (9)	385 (7)	201 (18)
No	5733 (86)	4936 (88)	797 (73)
Unknown	377 (5)	283 (5)	94 (9)

HIV status			
Positive	170 (2.5)	107 (2)	63 (6)
Negative	5526 (82.5)	4568 (81)	958 (88)
Unknown	1000 (15)	929 (17)	71 (7)
TB treatment history			
Yes	2033 (30)	1460 (26)	573 (53)
No	4657 (70)	4141 (74)	516 (47)

Baseline sputum			
AFB plus	540 (10)	2784 (50)	390 (36)
AFB minus	4171 (74)	2769 (49)	671 (61)
Unknown	893 (16)	51 (1)	31 (3)
Baseline culture			
Positive	4653 (83)	4653 (83)	
Negative	496 (9)	496 (9)	
Unknown	455 (8)	455 (8)	

GeneXpert result			
Positive	5457 (81.5)	4607 (82)	850 (78)
Negative	371 (5.5)	331 (6)	40 (4)
Unknown	868 (13)	666 (12)	202 (18)
Sputum at the end of treatment			
AFB plus	70 (1)	70 (1)	N/A
AFB minus	3739 (67)	3739 (67)	N/A
Unknown	1795 (32)	1795 (32)	N/A

Marital status			
Married	593 (54)	N/A	593 (54)
Single or other	469 (43)	N/A	469 (43)
Unknown	30 (3)	N/A	30 (3)
BMI category			
Underweight (<18.5)	222 (20)	N/A	222 (20)
Normal weight (18.5–24.9)	665 (61)	N/A	665 (61)

Overweight (25–29.9)	85 (8)	N/A	85 (8)
Obese (≥30)	23 (2)	N/A	23 (2)
Unknown	97 (9)	N/A	97 (9)
Diabetes			
Yes	107 (10)	N/A	107 (10)
No	269 (25)	N/A	269 (25)
Unknown	716 (65)	N/A	716 (65)

HCV status			
Positive	202 (19)	N/A	202 (19)
Negative	647 (59)	N/A	647 (59)
Unknown	243 (22)	N/A	243 (22)
Tobacco use			
Yes	617 (57)	N/A	617 (57)
No	103 (9)	N/A	103 (9)
Unknown	372 (34)	N/A	372 (34)

Alcohol intake			
None	602 (57)	N/A	602 (57)
Moderate	365 (34)	N/A	365 (34)
Excessive	97 (9)	N/A	97 (9)
Intravenous drug use			
Yes	38 (3)	N/A	38 (3)
No	860 (79)	N/A	860 (79)
Unknown	194 (18)	N/A	194 (18)

Resistance type			
Sensitive	71 (7)	N/A	71 (7)
RR/MDR	604 (55)	N/A	604 (55)
Pre-XDR	348 (32)	N/A	348 (32)
XDR	32 (3)	N/A	32 (3)
Unknown	37 (3)	N/A	37 (3)
Treatment outcome			
Favourable (cured + completed)	5169 (77)	4506 (80)	663 (61)
LFU	795 (12)	558 (10)	237 (22)
Died	302 (5)	241 (4)	61 (5.5)
Failure	278 (4)	185 (3)	93 (8.5)
Other	152 (2)	114 (2)	38 (3)

**Table 2.** Crude and adjusted association among bacteriologically confirmed pulmonary treatment episodes who were enrolled on first line drugs in NTP during 2015–2017 in the country of Georgia.

Characteristics	Total n=5403	cHR (95%CI)	aHR (95%CI)
Gender			
Male	4036	1.6 (1.3–2.0)	1.5 (1.2–2.0)
Female	1367	Ref	Ref
Age			
18–30	1185	Ref	Ref
31–45	1713	1.3 (1.0–2.6)	1.0 (0.8–1.2)
46–65	1900	0.9 (0.7–1.1)	0.6 (0.5–0.8)
>65	605	1.0 (0.7–1.4)	0.8 (0.6–1.1)

Region			
Tbilisi	1555	1.1 (0.9–1.4)	1.3 (1.1–1.6)
Other	3858	Ref	Ref
Employment			
Employed	621	Ref	Ref
Unemployed	4607	1.8 (1.3–2.5)	1.7 (1.2–2.3)

IDP history			
Yes	356	1.7 (1.3–2.3)	1.6 (1.2–2.2)
No	4765	Ref	Ref
Imprisonment history			
Yes	367	1.8 (1.3–2.3)	1.4 (1.0–1.8)
No	4785	Ref	Ref

HIV status			
Positive	98	1.3 (0.7–2.3)	1.2 (0.6–2.1)
Negative	4448	Ref	Ref
TB treatment history			
Yes	1395	2.6 (2.2–3.1)	2.3 (1.9–2.8)
No	4006	Ref	Ref

Baseline sputum			
AFB plus	2686	0.8 (0.7–1.0)	
AFB minus	2675	Ref	
Baseline culture			
Positive	4488	0.9 (0.7–1.2)	
Negative	484	Ref	

GeneXpert result			
Positive	4453	Ref	
Negative	318	0.9 (0.6–1.2)	
Sputum at the end of treatment			
AFB plus	68	4.5 (0.6–34.2)	
AFB minus	3689	Ref	

**Table 3.** Crude and adjusted association among bacteriologically confirmed pulmonary treatment episodes who were enrolled on second line drugs in NTP during 2015–2017 in the country of Georgia.

Characteristics	Total n=1017	cHR (95% CI)	aHR (95% CI)
Gender			
Male	821	2.3 (1.5–3.6)	2.0 (1.2–3.2)
Female	196	Ref	Ref
Age			
18–30	276	Ref	Ref
31–45	379	1.1 (0.8–1.6)	0.8 (0.6–1.1)
46–65	324	0.7 (0.5–1.1)	0.5 (0.3–0.8)
>65	38	0.8 (0.4–1.8)	0.7 (0.3–1.7)

Region			
Tbilisi	697	1.1 (0.8–1.4)	1.2 (0.8–1.6)
Other	320	Ref	Ref
Employment			
Employed	134	Ref	
Unemployed	838	1.4 (0.9–2.2)	

IDP history			
Yes	54	1.1 (0.6–2.1)	
No	789	Ref	
Imprisonment history			
Yes	180	1.6 (1.2–2.2)	1.1 (0.8–1.5)
No	752	Ref	Ref

HIV status			
Positive	50	2.0 (1.2–3.3)	1.7 (1.0–2.8)
Negative	900	Ref	Ref
TB treatment history			
Yes, first line drugs	246	1.5 (1.1–2.1)	1.4 (1.0–2.1)
Yes, second line drugs	275	2.0 (1.5–2.8)	2.2 (1.5–3.2)
No	493	Ref	Ref

Baseline culture			
Positive			
Negative			
GeneXpert result			
Positive	793	1.2 (0.5–2.7)	
Negative	37	Ref	

GeneXpert Rif resistance			
Susceptible	102	Ref	
Resistant	685	0.9 (0.6–1.4)	
Unknown	230		

Marital status			
Married	552	Ref	
Single or other	437	1.3 (1.0–1.6)	
Unknown			

BMI category			
Underweight (<18.5)	199	1.2 (0.8–1.6)	
Normal weight (18.5–24.9)	625	Ref	
Overweight (25–29.9)	81	0.9 (0.6–1.6)	
Obese (≥30)	23	0.4 (0.1–1.5)	
Unknown	89		

Diabetes			
Yes	101	0.5 (0.3–0.9)	0.7 (0.4–1.3)
No	247	Ref	Ref
Unknown	669		
HCV status			
Positive	202	2.0 (1.4–2.7)	1.6 (1.0–2.5)
Negative	475	Ref	Ref
Unknown	243		

Tobacco use			
Yes	570	1.3 (0.8–2.1)	
No	97	Ref	
Unknown	350		
Alcohol intake			
None	563	Ref	Ref
Moderate	335	1.4 (1.0–1.8)	1.3 (0.9–1.7)
Excessive	92	1.3 (0.8–2.1)	1.1 (0.7–1.8)

Intravenous drug use			
Yes	33	1.7 (0.9–3.2)	1.1 (0.6–2.2)
No	805	Ref	Ref
Unknown	179		
Resistance type			
Sensitive	68	Ref	Ref
RR/MDR	569	1.1 (0.6–2.0)	0.9 (0.5–1.6)
Pre-XDR	318	0.9 (0.5–1.7)	0.6 (0.3–1.1)
XDR	28	0.7 (0.2–2.0)	0.4 (0.1–1.2)
Unknown	34		

## References

[1] WHO. Global tuberculosis report 2019. Geneva: World Health Organization; 2020. Available from: http://www.who.int/tb/publications/global_report/en/

[2] WHO. Definitions and reporting framework for tuberculosis-2013 Revision; 2013. Accessed on: 2019 Nov 22. Available from: https://apps.who.int/iris/bitstream/handle/10665/79199/9789241505345_eng.pdf;jsessionid=A22FF4F90750CDEF883825A85CB3A48B?sequence=1

[3] WHO Regional Office for Europe. Tuberculosis surveillance and monitoring report in Europe 2020. Available from: https://www.euro.who.int/en/publications/abstracts/tuberculosis-surveillance-and-monitoring-report-in-europe-2020

[4] WHO. WHO Consolidated guidelines on drug-resistant tuberculosis treatment. 2019. Accessed on: 2019 Nov 22. Available from: https://www.who.int/tb/publications/2019/consolidated-guidelines-drug-resistant-TB-treatment/en/30946559

[5] Tupasi TE, Garfin AMCG, Kurbatova EV, Factors associated with loss to follow-up during treatment for multidrug-resistant tuberculosis, the Philippines, 2012–2014. Emerg Infect Dis 2016;22:491–502. 26889786 10.3201/eid2203.151788 PMC4766881

[6] Wohlleben J, Makhmudova M, Saidova F, Risk factors associated with loss to follow-up from tuberculosis treatment in Tajikistan: A case-control study. BMC Infect Dis 2017;17:543. 28778187 10.1186/s12879-017-2655-7 PMC5545046

[7] Schnaubelt ER, Charles M, Richard M, Loss to follow-up among patients receiving anti-tuberculosis treatment, Haiti, 2011–2015. Public Heal Action 2019;8:154–61. 10.5588/pha.18.0043 PMC6361484 30775274

[8] Kuchukhidze G, Kumar AMV, de Colombani P, Risk factors associated with loss to follow-up among multidrug-resistant tuberculosis patients in Georgia. Public Health Action 2014;4:S41–6. 26393097 10.5588/pha.14.0048 PMC4547510

[9] Adamashvili N, Kuchukhidze G, Baliashvili D, Long-term outcomes of patients lost to follow-up from multidrug-resistant tuberculosis treatment in the country of Georgia. Proceedings 48th World Conference on Lung Health, 2017, Guadalajara, Abstract SOA-415–13.

[10] World Bank. World Bank, Country Profile, Georgia. 2019. Available from: https://data.worldbank.org/country/georgia

[11] WHO. National Center for Tuberculosis and Lung Disease, Tbilisi, Georgia. Accessed on: 2019 Nov 22. Available from: https://www.who.int/workforcealliance/members_partners/member_list/tbgeo/en/.

[12] WHO. Tuberculosis Country Brief, 2016 Georgia. Accessed on: 2019 Nov 22. Available from: http://www.euro.who.int/en/health-topics/communicable-diseases/tuberculosis/publications/2017/tuberculosis-

[13] Javaid A, Shaheen Z, Shafqat M, Risk factors for high death and loss-to-follow-up rates among patients with multidrug-resistant tuberculosis at a programmatic management unit. Am J Infect Control 2017;45:190–3. 27769706 10.1016/j.ajic.2016.07.026

[14] Hirasen K, Berhanu R, Evans D, High rates of death and loss to follow-up by 12 months of rifampicin resistant TB treatment in South Africa. PLoS One 2018;13:e0205463. 30300403 10.1371/journal.pone.0205463 PMC6177165

[15] Mulongeni P, Hermans S, Caldwell J, HIV prevalence and determinants of loss-to-follow-up in adolescents and young adults with tuberculosis in Cape Town. PLoS One 2019;14:e0210937. 30721239 10.1371/journal.pone.0210937 PMC6363173

[16] Kliiman K, Altraja A. Predictors and mortality associated with treatment default in pulmonary tuberculosis. Int J Tuberc Lung Dis 2010;14:454–63. 20202304

[17] Kendall EA, Theron D, Franke MF, Alcohol, hospital discharge, and socioeconomic risk factors for default from multidrug resistant tuberculosis treatment in rural South Africa: a retrospective cohort study. PLoS One 2013;8:e83480. 24349518 10.1371/journal.pone.0083480 PMC3862731

